# Supplementary figures and images for: Impacts of climate change on agro-climatic suitability of major food crops in Ghana
Source: PLoS One. 2020 Jun 29;15(6):e0229881. doi: 10.1371/journal.pone.0229881 (PMC7323970; doi:10.1371/journal.pone.0229881)

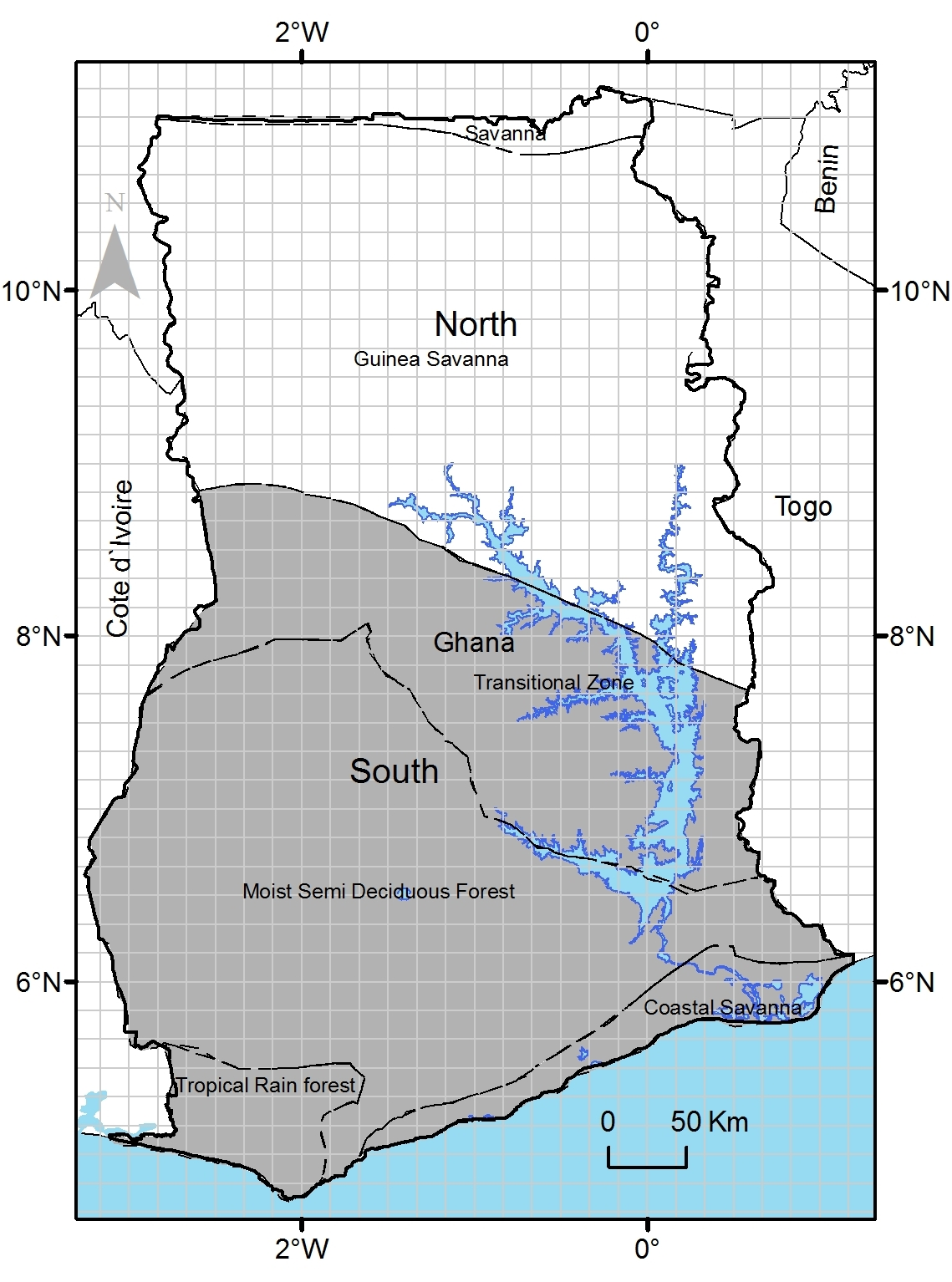

Supplement: S1 Fig — (TIF) [file pone.0229881.s001.tif]
