# Supplementary material for: Impacts of climate change on agro-climatic suitability of major food crops in Ghana
Source: PLoS One. 2020 Jun 29;15(6):e0229881. doi: 10.1371/journal.pone.0229881 (PMC7323970; doi:10.1371/journal.pone.0229881)
Supplement: S1 Table — (DOCX) [file pone.0229881.s002.docx]

**Tables**

**S1 Table: Accuracy metrics for modelling maize, sorghum, groundnut and cassava suitability in Ghana under current climate conditions.**

| **Crop** | **Maize** | | | | **Sorghum** | | | |
| --- | --- | --- | --- | --- | --- | --- | --- | --- |
| Metrics/Class | Limited | Marginal | Suitable | High | Limited | Marginal | Suitable | High |
| Sensitivity | 0.67 | 0.75 | 0.83 | 0.74 | 0.91 | 0.90 | 0.71 | 0.66 |
| Specificity | 0.93 | 0.89 | 0.88 | 0.95 | 0.92 | 0.95 | 0.94 | 0.95 |
| Positive Pred Value | 0.74 | 0.77 | 0.70 | 0.79 | 0.86 | 0.88 | 0.71 | 0.76 |
| Negative Pred Value | 0.90 | 0.88 | 0.94 | 0.94 | 0.96 | 0.96 | 0.94 | 0.91 |
| Precision | 0.74 | 0.77 | 0.70 | 0.79 | 0.86 | 0.88 | 0.71 | 0.76 |
| Recall | 0.67 | 0.75 | 0.83 | 0.74 | 0.91 | 0.90 | 0.71 | 0.66 |
| F1-score | 0.70 | 0.76 | 0.76 | 0.76 | 0.89 | 0.89 | 0.71 | 0.70 |
| Prevalence | 0.23 | 0.32 | 0.25 | 0.19 | 0.34 | 0.28 | 0.17 | 0.21 |
| Detection Rate | 0.15 | 0.24 | 0.21 | 0.14 | 0.31 | 0.25 | 0.12 | 0.14 |
| Detection Prevalence | 0.21 | 0.31 | 0.30 | 0.18 | 0.36 | 0.29 | 0.17 | 0.18 |
| Balanced Accuracy | 0.80 | 0.82 | 0.85 | 0.85 | 0.92 | 0.92 | 0.82 | 0.80 |
|  |  | | | |  | | | |
|  | **Groundnut** | | | | **Cassava** | | | |
| Metrics/Class | Limited | Marginal | Suitable | High | Limited | Marginal | Suitable | High |
| Sensitivity | 0.86 | 0.87 | 0.61 | 0.66 | 0.73 | 0.73 | 0.64 | 0.71 |
| Specificity | 0.90 | 0.88 | 0.91 | 0.97 | 0.95 | 0.86 | 0.88 | 0.91 |
| Positive Pred Value | 0.74 | 0.70 | 0.71 | 0.88 | 0.77 | 0.77 | 0.61 | 0.63 |
| Negative Pred Value | 0.95 | 0.95 | 0.86 | 0.90 | 0.93 | 0.82 | 0.90 | 0.94 |
| Precision | 0.74 | 0.70 | 0.71 | 0.88 | 0.77 | 0.77 | 0.61 | 0.63 |
| Recall | 0.86 | 0.87 | 0.61 | 0.66 | 0.73 | 0.73 | 0.64 | 0.71 |
| F1-score | 0.80 | 0.78 | 0.66 | 0.75 | 0.75 | 0.75 | 0.62 | 0.67 |
| Prevalence | 0.25 | 0.25 | 0.27 | 0.23 | 0.20 | 0.40 | 0.22 | 0.17 |
| Detection Rate | 0.21 | 0.22 | 0.17 | 0.15 | 0.15 | 0.29 | 0.14 | 0.12 |
| Detection Prevalence | 0.28 | 0.31 | 0.23 | 0.17 | 0.19 | 0.38 | 0.23 | 0.20 |
| Balanced Accuracy | 0.88 | 0.87 | 0.76 | 0.82 | 0.84 | 0.79 | 0.76 | 0.81 |
