# Supplementary material for: Impacts of climate change on agro-climatic suitability of major food crops in Ghana
Source: PLoS One. 2020 Jun 29;15(6):e0229881. doi: 10.1371/journal.pone.0229881 (PMC7323970; doi:10.1371/journal.pone.0229881)
Supplement: S2 Table — (DOCX) [file pone.0229881.s003.docx]

**S4 Table: Percentage of dual crop suitability under each climatic condition in Ghana derived from combining the suitability levels of each of the two crops**

| Combinations | Scenario | Limited-Limited | | Limited-Marginal | | marginal-Marginal | | Marginal-Moderate | | Moderate-Moderate | | Moderate-High | | High-High | |
| --- | --- | --- | --- | --- | --- | --- | --- | --- | --- | --- | --- | --- | --- | --- | --- |
|  |  | % | Area(km2) | % | Area(km2) | % | Area(km2) | % | Area(km2) | % | Area(km2) | % | Area(km2) | % | Area(km2) |
| Maize - Sorghum | Current | 8.3 | 19931 | 12.6 | 30065 | 20.8 | 49658 | 21.5 | 51347 | 20.3 | 48644 | 12.9 | 30741 | 3.7 | 8783 |
|  | R26 | 4.0 | 9459 | 8.3 | 19931 | 25.3 | 60468 | 31.8 | 76007 | 23.3 | 55738 | 6.8 | 16215 | 0.6 | 1351 |
|  | R85 | 4.7 | 11148 | 10.2 | 24322 | 27.4 | 65535 | 30.1 | 71953 | 21.0 | 50333 | 5.8 | 13850 | 0.8 | 2027 |
| Maize - Cassava | Current | 10.0 | 23984 | 15.0 | 35808 | 25.8 | 61819 | 23.7 | 56752 | 12.9 | 30741 | 7.3 | 17566 | 5.2 | 12499 |
|  | R26 | 7.3 | 17566 | 17.4 | 41550 | 27.8 | 66548 | 20.9 | 49996 | 16.9 | 40537 | 7.6 | 18242 | 2.0 | 4729 |
|  | R85 | 7.8 | 18579 | 17.1 | 40875 | 28.4 | 67899 | 23.9 | 57090 | 16.5 | 39524 | 4.8 | 11485 | 1.6 | 3716 |
| Maize - Groundnut | Current | 3.0 | 7094 | 16.8 | 40199 | 16.5 | 39524 | 32.8 | 78371 | 20.3 | 48644 | 5.1 | 12161 | 5.5 | 13175 |
|  | R26 | 0.3 | 676 | 10.2 | 24322 | 25.4 | 60805 | 38.4 | 91884 | 20.2 | 48307 | 5.4 | 12837 | 0.1 | 338 |
|  | R85 | 4.2 | 10134 | 5.4 | 12837 | 30.5 | 72967 | 25.3 | 60468 | 23.0 | 55063 | 10.0 | 23984 | 1.6 | 3716 |
| Cassava - Sorghum | Current | 8.3 | 19931 | 14.8 | 35470 | 28.1 | 67224 | 20.9 | 49996 | 18.1 | 43239 | 4.4 | 10472 | 5.4 | 12837 |
|  | R26 | 2.7 | 6418 | 15.1 | 36145 | 36.7 | 87830 | 24.3 | 58103 | 16.7 | 39861 | 2.8 | 6756 | 1.7 | 4054 |
|  | R85 | 3.2 | 7770 | 17.1 | 40875 | 36.0 | 86141 | 25.4 | 60805 | 14.1 | 33781 | 3.0 | 7094 | 1.1 | 2702 |
| Cassava - Groundnut | Current | 5.5 | 13175 | 14.3 | 34119 | 27.1 | 64859 | 28.7 | 68575 | 15.1 | 36145 | 8.9 | 21282 | 0.4 | 1013 |
|  | R26 | 2.3 | 5405 | 17.7 | 42226 | 28.1 | 67224 | 34.6 | 82763 | 12.1 | 29051 | 5.2 | 12499 | 0.0 | 0 |
|  | R85 | 10.6 | 25336 | 11.3 | 27025 | 19.8 | 47293 | 26.1 | 62494 | 26.6 | 63508 | 5.5 | 13175 | 0.1 | 338 |
| Groundnut-Sorghum | Current | 13.6 | 32430 | 11.7 | 28038 | 13.4 | 32092 | 19.4 | 46280 | 19.1 | 45604 | 19.4 | 46280 | 3.5 | 8445 |
|  | R26 | 5.9 | 14188 | 15.5 | 37159 | 20.1 | 47969 | 24.9 | 59454 | 17.4 | 41550 | 13.6 | 32430 | 2.7 | 6418 |
|  | R85 | 0.1 | 338 | 4.1 | 9796 | 32.1 | 76682 | 37.3 | 89181 | 20.6 | 49320 | 4.9 | 11823 | 0.8 | 2027 |
