# Supplementary material for: Impacts of climate change on agro-climatic suitability of major food crops in Ghana
Source: PLoS One. 2020 Jun 29;15(6):e0229881. doi: 10.1371/journal.pone.0229881 (PMC7323970; doi:10.1371/journal.pone.0229881)
Supplement: S3 Table — (DOCX) [file pone.0229881.s004.docx]

**S3 Table: Assessment of diversification potential for the north and south of Ghana using pairwise comparisons.**

**D1:** Cassava-Groundnut

| Cassava-Groundnut | Current | | | | RCP2.6 | | | | RCP8.5 | | | |
| --- | --- | --- | --- | --- | --- | --- | --- | --- | --- | --- | --- | --- |
|  | South | | North | | South | | North | | South | | North | |
|  | % | Area(km2) | % | Area(km2) | % | Area(km2) | % | Area(km2) | % | Area(km2) | % | Area(km2) |
| Limited-Limited | 1.5 | 3700 | 3.8 | 9082 | 0.4 | 1009 | 1.7 | 4037 | 0.1 | 336 | 10.4 | 24892 |
| Limited-Marginal | 5.3 | 12783 | 9.1 | 21865 | 5.3 | 12783 | 12.4 | 29602 | 1.7 | 4037 | 9.6 | 22874 |
| Marginal-Marginal | 16.6 | 39693 | 10.5 | 25229 | 13.9 | 33302 | 14.2 | 33975 | 11.4 | 27247 | 8.7 | 20856 |
| Marginal-Moderate | 15.3 | 36666 | 13.4 | 31956 | 23.3 | 55840 | 11.4 | 27247 | 18.7 | 44739 | 7.3 | 17492 |
| Moderate-Moderate | 11.0 | 26238 | 4.1 | 9755 | 10.7 | 25565 | 1.4 | 3364 | 21.1 | 50457 | 5.3 | 12783 |
| Moderate-High | 8.3 | 19847 | 0.6 | 1346 | 4.8 | 11437 | 0.4 | 1009 | 5.3 | 12783 | 0.1 | 336 |
| High-High | 0.4 | 1009 | 0.0 | 0 | 0.0 | 0 | 0.0 | 0 | 0.1 | 336 | 0.0 | 0 |

**D2:** Cassava – Sorghum

| Cassava - Sorghum | Current | | | | RCP2.6 | | | | RCP8.5 | | | |
| --- | --- | --- | --- | --- | --- | --- | --- | --- | --- | --- | --- | --- |
|  | South | | North | | South | | North | | South | | North | |
|  | % | Area(km2) | % | Area(km2) | % | Area(km2) | % | Area(km2) | % | Area(km2) | % | Area(km2) |
| Limited-Limited | 3.4 | 8073 | 5.1 | 12110 | 1.7 | 4037 | 5.1 | 12110 | 1.7 | 4037 | 1.1 | 2691 |
| Limited-Marginal | 8.3 | 19847 | 6.5 | 15474 | 8.3 | 19847 | 6.5 | 15474 | 6.5 | 15474 | 8.7 | 20856 |
| Marginal-Marginal | 14.1 | 33638 | 13.9 | 33302 | 14.1 | 33638 | 13.9 | 33302 | 17.2 | 41039 | 19.3 | 46084 |
| Marginal-Moderate | 10.7 | 25565 | 10.1 | 24220 | 10.7 | 25565 | 10.1 | 24220 | 16.6 | 39693 | 7.6 | 18165 |
| Moderate-Moderate | 13.5 | 32293 | 4.8 | 11437 | 13.5 | 32293 | 4.8 | 11437 | 12.7 | 30274 | 4.2 | 10091 |
| Moderate-High | 3.7 | 8746 | 0.7 | 1682 | 3.7 | 8746 | 0.7 | 1682 | 2.4 | 5719 | 0.4 | 1009 |
| High-High | 4.9 | 11773 | 0.4 | 1009 | 4.9 | 11773 | 0.4 | 1009 | 1.5 | 3700 | 0.1 | 336 |

**D3:** Groundnut-Sorghum

| Groundnut-Sorghum | Current | | | | RCP2.6 | | | | RCP8.5 | | | |
| --- | --- | --- | --- | --- | --- | --- | --- | --- | --- | --- | --- | --- |
|  | South | | North | | South | | North | | South | | North | |
|  | % | Area(km2) | % | Area(km2) | % | Area(km2) | % | Area(km2) | % | Area(km2) | % | Area(km2) |
| Limited-Limited | 10.3 | 24556 | 3.4 | 8073 | 4.9 | 11773 | 1.0 | 2355 | 0.0 | 0 | 0.1 | 336 |
| Limited-Marginal | 9.3 | 22201 | 2.5 | 6055 | 12.0 | 28593 | 3.8 | 9082 | 1.3 | 3027 | 2.8 | 6728 |
| marginal-Marginal | 12.1 | 28929 | 1.1 | 2691 | 16.0 | 38348 | 3.8 | 9082 | 19.0 | 45412 | 13.2 | 31620 |
| Marginal-Moderate | 7.5 | 17828 | 12.0 | 28593 | 11.3 | 26911 | 13.5 | 32293 | 27.1 | 64922 | 10.0 | 23883 |
| Moderate-Moderate | 10.3 | 24556 | 8.7 | 20856 | 6.5 | 15474 | 10.8 | 25901 | 11.0 | 26238 | 9.8 | 23547 |
| Moderate-High | 8.7 | 20856 | 10.7 | 25565 | 7.9 | 18837 | 5.9 | 14128 | 0.1 | 336 | 4.6 | 11101 |
| High-High | 0.4 | 1009 | 3.1 | 7400 | 0.0 | 0 | 2.7 | 6391 | 0.0 | 0 | 0.8 | 2018 |

**D4:** Maize-Cassava

| Maize-Cassava | Current | | | | RCP2.6 | | | | RCP8.5 | | | |
| --- | --- | --- | --- | --- | --- | --- | --- | --- | --- | --- | --- | --- |
|  | South | | North | | South | | North | | South | | North | |
|  | % | Area(km2) | % | Area(km2) | % | Area(km2) | % | Area(km2) | % | Area(km2) | % | Area(km2) |
| Limited-Limited | 1.8 | 4373 | 8.3 | 19847 | 1.5 | 3700 | 5.9 | 14128 | 2.7 | 6391 | 0.6 | 1346 |
| Limited-Marginal | 3.4 | 8073 | 11.5 | 27583 | 1.7 | 4037 | 15.6 | 37338 | 9.7 | 23210 | 7.0 | 16819 |
| Marginal-Marginal | 13.4 | 31956 | 12.5 | 29938 | 12.5 | 29938 | 15.5 | 37002 | 9.0 | 21528 | 7.5 | 17828 |
| Marginal-Moderate | 14.8 | 35320 | 9.0 | 21528 | 16.9 | 40366 | 3.9 | 9419 | 18.8 | 45075 | 13.8 | 32965 |
| Moderate-Moderate | 12.7 | 30274 | 0.1 | 336 | 16.3 | 39020 | 0.6 | 1346 | 12.9 | 30947 | 7.5 | 17828 |
| Moderate-High | 7.3 | 17492 | 0.0 | 0 | 7.6 | 18165 | 0.0 | 0 | 2.5 | 6055 | 2.5 | 6055 |
| High-High | 5.2 | 12446 | 0.0 | 0 | 2.0 | 4709 | 0.0 | 0 | 2.8 | 6728 | 2.7 | 6391 |

**D5:** Maize- Groundnut

| Maize- Groundnut | Current | | | | RCP2.6 | | | | RCP8.5 | | | |
| --- | --- | --- | --- | --- | --- | --- | --- | --- | --- | --- | --- | --- |
|  | South | | North | | South | | North | | South | | North | |
|  | % | Area(km2) | % | Area(km2) | % | Area(km2) | % | Area(km2) | % | Area(km2) | % | Area(km2) |
| Limited-Limited | 2.7 | 6391 | 0.6 | 1346 | 0.3 | 673 | 0.0 | 0 | 0.0 | 0 | 4.2 | 10091 |
| Limited-Marginal | 9.7 | 23210 | 7.0 | 16819 | 6.3 | 15137 | 3.9 | 9419 | 0.1 | 336 | 5.2 | 12446 |
| Marginal-Marginal | 9.0 | 21528 | 7.5 | 17828 | 11.4 | 27247 | 13.9 | 33302 | 14.8 | 35320 | 16.2 | 38684 |
| Marginal-Moderate | 18.8 | 45075 | 13.8 | 32965 | 21.5 | 51467 | 17.0 | 40702 | 20.8 | 49785 | 4.4 | 10428 |
| Moderate-Moderate | 12.9 | 30947 | 7.5 | 17828 | 15.6 | 37338 | 4.5 | 10764 | 15.9 | 38011 | 6.9 | 16483 |
| Moderate-High | 2.5 | 6055 | 2.5 | 6055 | 3.4 | 8073 | 2.0 | 4709 | 5.3 | 12783 | 4.6 | 11101 |
| High-High | 2.8 | 6728 | 2.7 | 6391 | 0.0 | 0 | 0.1 | 336 | 1.5 | 3700 | 0.0 | 0 |

**D6:** Maize – Sorghum

| Maize - Sorghum | Current | | | | RCP2.6 | | | | RCP8.5 | | | |
| --- | --- | --- | --- | --- | --- | --- | --- | --- | --- | --- | --- | --- |
|  | South | | North | | South | | North | | South | | North | |
|  | % | Area(km2) | % | Area(km2) | % | Area(km2) | % | Area(km2) | % | Area(km2) | % | Area(km2) |
| Limited-Limited | 7.5 | 17828 | 1.1 | 2691 | 3.9 | 9419 | 0.3 | 673 | 4.8 | 11437 | 0.1 | 336 |
| Limited-Marginal | 8.7 | 20856 | 3.8 | 9082 | 4.6 | 11101 | 3.7 | 8746 | 6.5 | 15474 | 3.7 | 8746 |
| Marginal-Marginal | 7.5 | 17828 | 13.2 | 31620 | 11.4 | 27247 | 13.8 | 32965 | 13.5 | 32293 | 13.8 | 32965 |
| Marginal-Moderate | 11.4 | 27247 | 10.0 | 23883 | 20.1 | 48103 | 11.4 | 27247 | 16.7 | 40030 | 13.1 | 31284 |
| Moderate-Moderate | 13.2 | 31620 | 7.0 | 16819 | 14.8 | 35320 | 8.7 | 20856 | 13.2 | 31620 | 8.0 | 19174 |
| Moderate-High | 8.9 | 21192 | 4.1 | 9755 | 3.2 | 7737 | 3.5 | 8410 | 3.8 | 9082 | 2.0 | 4709 |
| High-High | 1.4 | 3364 | 2.3 | 5382 | 0.4 | 1009 | 0.1 | 336 | 0.0 | 0 | 0.8 | 2018 |
